# Stereoscopic Segmentation Cues Improve Visual Timing Performance in Spatiotemporally Cluttered Environments

**DOI:** 10.1177/2041669517699222

**Published:** 2017-04-03

**Authors:** Daniel Talbot, Erik Van der Burg, John Cass

**Affiliations:** School of Social Science and Psychology, Western Sydney University, Australia; Department of Experimental and Applied Psychology, Vrije Universiteit Amsterdam, The Netherlands; School of Psychology, University of Sydney, Australia; School of Social Science and Psychology, Western Sydney University, Australia

**Keywords:** binocular vision, temporal processing, temporal selection or modulation, time perception

## Abstract

Recently, Cass and Van der Burg demonstrated that temporal order judgment (TOJ) precision could be profoundly impaired by the mere presence of dynamic visual clutter elsewhere in the visual field. This study examines whether presenting target and distractor objects in different depth planes might ameliorate this remote temporal camouflage (RTC) effect. TOJ thresholds were measured under static and dynamic (flickering) distractor conditions. In Experiment 1, targets were presented at zero, crossed, or uncrossed disparity, with distractors fixed at zero disparity. Thresholds were significantly elevated under dynamic compared with static contextual conditions, replicating the RTC effect. Crossed but not uncrossed disparity targets improved performance in dynamic distractor contexts, which otherwise produce substantial RTC. In Experiment 2, the assignment of disparity was reversed: targets fixed at zero disparity; distractors crossed, uncrossed, or zero. Under these conditions, thresholds improved significantly in the nonzero distractor disparity conditions. These results indicate that presenting target and distractor objects in different planes can significantly improve TOJ performance in dynamic conditions. In Experiment 3, targets were each presented with a different sign of disparity (e.g., one crossed and the other uncrossed), with no resulting performance benefits. Results suggest that disparity can be used to alleviate the performance-diminishing effects of RTC, but only if both targets constitute a single and unique disparity-defined surface.

## Introduction

One of the primary functions of stereopsis is to facilitate object segmentation, particularly in cluttered visual environments (Nakayama & Silverman, 1986). One way stereopsis accomplishes this is by alleviating contextual effects such as crowding and surround suppression, which may otherwise interfere with visual performance (Astle, McGovern, & McGraw, 2014; Kooi, Toet, Tripathy, & Levi, 1994; Petrov, Carandini, & McKee, 2005; Wardle, Cass, Brooks & Alais, 2010).

Recently, Cass and Van der Burg (2014) reported a new contextual phenomenon in which visual timing performance (temporal order judgment [TOJ] thresholds) is profoundly disrupted when the target objects were surrounded by irrelevant luminance modulating objects elsewhere in the visual field compared with when these irrelevant objects were static (i.e., not modulating). This phenomenon, known as remote temporal camouflage (RTC) differs in several key respects to other previously reported contextual phenomena, such as crowding, surround suppression, and motion-induced blindness (Bonneh, Cooperman, & Sagi, 2001; Pelli, & Tillman, 2008; Petrov et al., 2005; Petrov & McKee, 2006; Wallis, & Arnold, 2009). Notably, it operates over a far greater spatial extent and is relatively resistant to segmentation cues such as colour, which might otherwise improve performance (Kooi et al., 1994).

The causes of RTC are currently unknown. Based on the available evidence, it is plausible that RTC may result from long-range motion masking. According to this view, TOJs are assumed to be based on the perceived direction of motion of the target events (long-range first-order motion). The presence of dynamic distractors then introduces irrelevant motion signals to this direction decision, thereby masking the target-relevant motion signal.

Remarkably, the RTC-related increase in TOJ thresholds, from approximately 20 ms in conditions with static (nondynamic) distractors to more than 80 ms in dynamic distractor conditions, corresponds closely with Holcombe’s (2009) dual speed limit scheme of human visual performance. According to this scheme, *lower-order* motion discrimination tasks are performed with high temporal precision (thresholds ∼20 ms) and *higher-order* tasks afford relatively poor temporal precision (thresholds > 80 ms). It is conceivable, therefore, that the threshold elevation accompanying RTC represents a qualitative shift in the nature of the TOJ task: from judgments based on *lower-order* (possibly preattentive) motion discrimination in the context of nondynamic distractor environments to judgments based on attentionally demanding *higher-order* motion discrimination in dynamic distractor environments.

Given that introducing disparity between targets and their immediate contexts has been found to assuage deleterious contextual effects involving nontemporal tasks (e.g., detection, orientation discrimination, and visual search), it seems reasonable to expect that analogous performance benefits may be conferred to TOJ tasks performed in dynamic distractor environments. However, given that RTC is relatively immune from the performance benefits that might otherwise be expected from strong segmentation cues such as colour (Cass & Van der Burg, 2014), it is conceivable that disparity-defined segmentation cues may similarly fail to confer performance advantages.

This article explores whether introducing different binocular disparity signals between target and distractor events affects TOJ performance measured in dynamic and nondynamic (static) distractor contexts. In Experiment 1, distractors are presented in the zero disparity plane with both targets assigned crossed, uncrossed, or zero disparity. This situation is reversed in Experiment 2, with the targets fixed at zero disparity and distractors all assigned crossed, uncrossed, or zero disparity. Finally, Experiment 3 explores the role of relative and absolute disparity differences by presenting a given pair of target events in different depth planes (crossed and uncrossed disparity) and fixing distractors at zero disparity.

## General Methods

### Observers

Sixteen observers were recruited for Experiment 1. Two observers were omitted from the study, as they were unable to fuse the stimulus through the stereoscope. One observer was omitted through data screening due to poor performance on the Fly Stereo Acuity Test, (FSAT; Vision Assessment Corporation, Elk Grove Village, IL, USA), reliably identifying only three of graded circle targets (200 seconds of arc). All other observers reliably identified at least six of the targets (corresponding to 80 seconds of arc). Eleven observers were recruited for Experiments 2 and 3. All observers had normal or corrected-to-normal visual acuity. All but two of the observers (authors D. T. and J. C.) were naïve to the purposes of the experiments. These authors participated in each experiment. Experimental protocol was approved by Western Sydney University’s Human Research and Ethics Committee, approval number H8862.

### Stimuli

Stimuli were displayed in a darkened room on an LCD monitor (Samsung s27a950d; screen dimensions = 600 × 350 mm; 1024 × 768 pixels, 85 Hz). The background colour was grey and held at a constant luminance of 32 cd/m^2^ for all experiments. Observers viewed the stimuli through a stereoscope constructed using two pairs of front-surface mirrors. A forehead rest was used to hold the observers’ head in correct position so that their face was held in the same fronto-parallel plane as the monitor display and the position of their two eyes were parallel with the top and bottom edge of the monitor. Viewing distance including the stereoscopic path was 600 mm. A septum was placed between each set of left- and right-eyed mirrors to preclude interocular contamination. The mirrors in the stereoscope were individually aligned for each observer to ensure sufficient fusional range was available.

Target stimuli were vertically arranged pairs of black disks (diameter = 1.5° of visual angle). Each pair of target disks was separated by 220 pixels (separated by 6.15°) along the *y* axis, with each pair presented to each eye. Sixteen distractor disks (diameter = 1.5° of visual angle; eight presented to each eye) were arranged in pairs above and below each target, respectively. Each pair of distractors was separated from the target by 0.2° on the *y* axis. The *y*-axis position of each of the 16 distractor disk arrays was stable throughout the experiments. To achieve binocular disparity, the relative positions of the target disks varied along the *x* axis. All stimuli contained two black and white fusion lock frames surrounding targets and distractors in order to consolidate stereoscopic fusion. The dimensions of both frames were 134 × 405 pixels, positioned centrally around each fixation dot, respectively.

**Figure 1.**
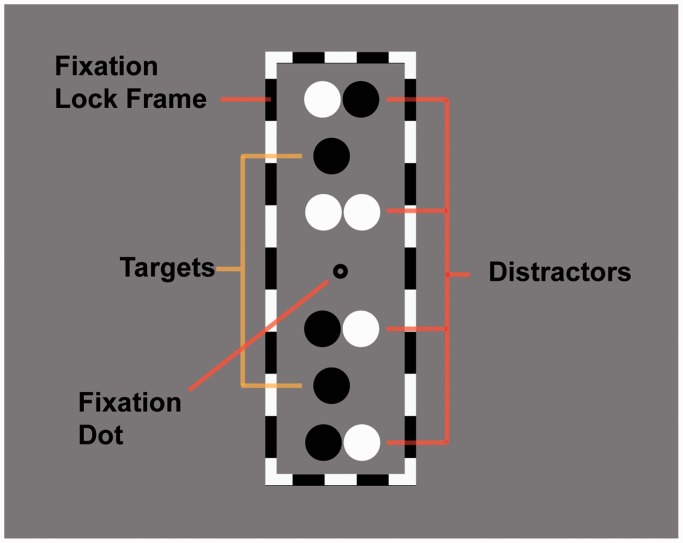
Example of how the stimulus would be perceived when binocularly fused.

**Figure 2.**
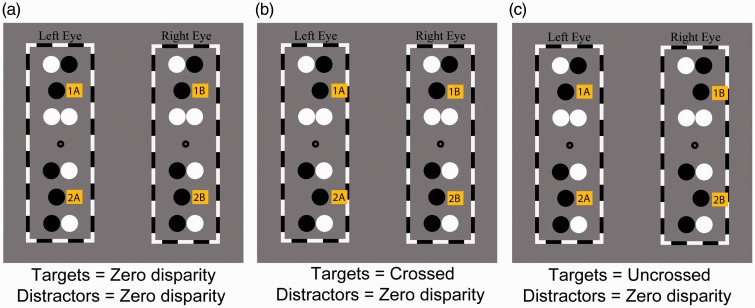
(a) to (c) Example stimuli representing each target disparity condition used in Experiment 1. Distractors have zero disparity in all conditions. (a) Zero disparity targets. (b) Crossed disparity targets. (c) Uncrossed disparity targets. 1A and 1B, fused top target; 2A and 2B, fused bottom target.

The stimulus was viewed through a stereoscope, which enabled observers to binocularly fuse each eye’s image of the stimulus and thereby perceive a single fixation dot, two target disks (at a particular depth depending upon the binocular disparity condition), eight distractor disks (all at zero disparity [Experiments 1 and 3]), or the converse (Experiment 2), and one black and white frame. An example of the fused stimulus as viewed by observers is shown in Figure 1. Stimuli for disparity condition (zero, crossed, and uncrossed disparity) is shown in Figure 2.

Nonzero disparities used in these experiments were fixed at 800 arc seconds (0.22 cm). Perceived depth between target disks and distractor disks was calculated using the following equation: $\mathrm{pd}=\frac{\text{z}}{(\frac{\text{E}}{\text{d}})-1}$, where *pd* = perceived depth, viewing distance (*Z*) = 60 cm, interocular separation (*E*) = 6.5 cm, and screen disparity (*d*) = .22 cm. The perceived depth of disks presented with nonzero disparity was calculated to be 2.12 cm. Targets with zero, crossed, and uncrossed disparity, respectively, are shown in Figure 2.

Finally, each observer completed the FSAT in order to assess stereo depth perception sensitivity (Vision Assessment Corporation, Elk Grove Village, IL, USA). The FSAT produces a score out of 10, with higher scores indicating higher sensitivity to stereoscopic disparity. Each observer viewed the FSAT stimuli through polarising lenses which enabled separate images to be presented to each eye. As our experiments investigate the effect of stereoscopic cues on performance, FSAT scores used to screen the sample for observers with insufficient stereo depth perception sensitivity (a score below 6; Vision Assessment Corporation, Elk Grove Village, IL, USA).

### Procedure

All observers completed a practice session consisting of 24 trials to familiarise them with the task. Targets and distractors were presented independently to each eye using the stereoscope. Each trial began with a singular black fixation dot presented at the centre of the screen for 500 ms. Observers were instructed to maintain fixation upon this point for the duration of each experimental trial and were informed that they could move their eyes between trials should they feel fatigued. The two black target disks and distractors appeared above and below the fixation dot (separated by 6.15**°**) at their disparity-linked *x*-coordinates specified earlier.

In Experiment 1, three different target disparities were used: uncrossed, zero, and crossed. Distractors were fixed at zero disparity. In Experiment 2, this situation was reversed: targets fixed at zero disparity, and distractors all assigned crossed, zero, or uncrossed disparity. Finally, Experiment 3 employed three different target disparity configurations: top target crossed or bottom target uncrossed, zero, and bottom target crossed or top target uncrossed disparities, and presented all distractors in the zero disparity plane. Regardless of target or distractor disparity, during the course of a given trial, the luminance polarity of the distractors either modulated abruptly across time (dynamic condition) or remained unchanged throughout the trial (static condition). Hence, each experiment employed a 3 × 2 (3 levels of disparity × 2 levels of distractor dynamics) within-subjects design.

**Figure 3.**
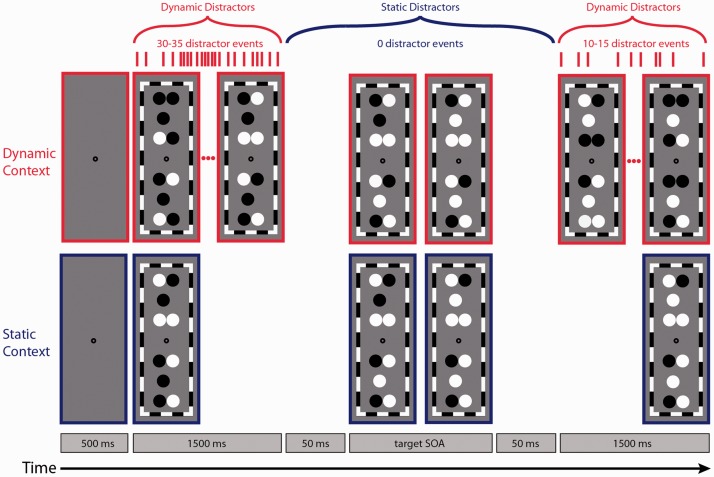
Example trial sequence representing distractor contextual conditions: dynamic distractors (red) and static distractors (blue). Each panel depicts a cyclopean simulation of the stimulus.

A pilot study measured TOJ thresholds without any distractors at each level of target disparity used in Experiment 1 and found no variation in performance across the various levels of disparity relative to any static distractor condition, replicating Cass and Van der Burg (2014). For reasons of brevity, we have therefore chosen to omit this *Targets Alone* condition from the remainder of this manuscript. In the dynamic distractor condition, each distractor disk (as specified earlier) was assigned to be either black (2 cd/m^2^) or white (62 cd/m^2^) at the beginning of each trial. A dynamic distractor trial consisted of three sets of luminance changes. The initial set involved a randomly determined number of distractor disks (out of the possible eight) abruptly changing luminance polarity (from white to black or vice versa) every 50 ms until a number of randomly determined events had occurred (30–35). Then, after period of 50 ms in which no changes occurred, the second set of changes was initiated. In this set, the luminance of one of the two target disks changed abruptly from black to white (62 cd/m^2^) followed by an equivalent luminance change in the other target after a randomly determined stimulus onset asynchrony (SOA; −400, −200, −93, −67, −27, −13, 13, 27, 67, 93, 200, 400 ms). Negative SOAs indicate that the luminance of the bottom target changed first, whereas positive SOAs indicate that the luminance of the top target changed first. The third set of changes began 50 ms after the onset of the second target disk change. This set of distractor changes was identical in procedure to the first set of distractor changes, except the number of changes was selected randomly between (10 and 15). In the static condition, the luminance of each distractor was randomly determined prior to each experimental trial and remained constant throughout the trial. Aside from the distractor changes, the timing of the static condition was identical to the dynamic condition. On each trial, after a key-press response, the display turned grey for 500 ms, then the next trial began. The observers’ task was to determine whether the bottom or top target luminance change occurred first. They indicated bottom or top first by pressing the *N* key or *J* key, respectively. Figure 3 shows an example trial sequence representing the two distractor conditions. Note that the temporal and luminance properties of each target and distractor in the corresponding left and right eye images were identical.

Just noticeable differences (JNDs) were obtained in each condition by fitting a cumulative Gaussian separately to each observer’s data (proportion of *top first* responses as a function of target SOA) using a Levenburg-Marquardt algorithm maximum likelihood fitting procedure and multiplying the fitted standard deviation of this fit by 0.67 (Cass & Van der Burg, 2014).

Experiment 1 involved testing both static and dynamic distractor conditions at each level of target disparity using a blocked design, with each block corresponding to a particular level of target disparity (uncrossed, zero, and crossed) and distractor dynamics (static or dynamic context), yielding six blocks in total. This sequence was randomly counterbalanced across observers. Each block consisted of 120 trials, yielding a total of 720 trials per observer. Experiments 2 and 3 were identical in most respects to Experiment 1, with the exceptions that in Experiment 2, the disparity of the distractor object was manipulated rather than the targets; and in Experiment 3, each target was assigned an equal magnitude of disparity but opposite sign (top crossed or bottom uncrossed, or top uncrossed or bottom crossed).

## Experiment 1

### Results and Discussion

**Figure 4.**
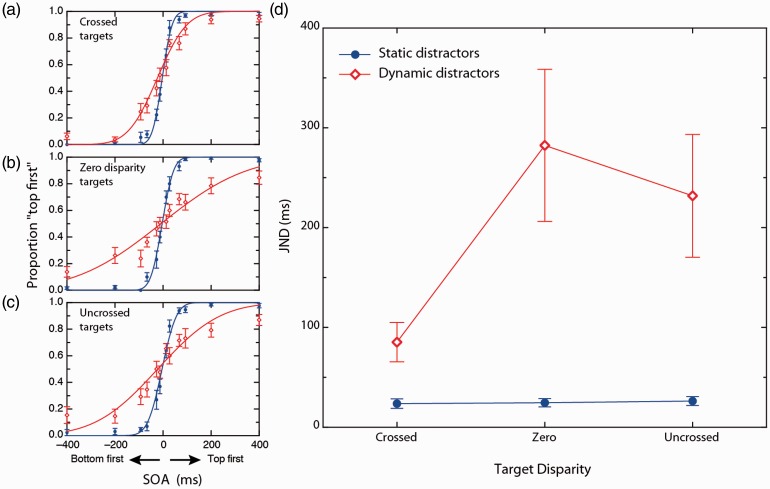
(a) to (c) Effect of target disparity and distractor dynamics on performance at each level of target SOA averaged across observers. Red and blue symbols and curves were measured under dynamic and static distractor conditions, respectively. (a) Crossed disparity targets, (b) zero disparity targets, and (c) uncrossed disparity targets. Curves shown are cumulative Gaussian fits of observer-averaged data and are shown here for illustrative purposes only. Statistical analyses were based on fits of individual observers. (d) Mean JNDs derived under static and dynamic contextual conditions, for crossed, zero, and uncrossed target disparities. Note that the distractors were fixed at zero disparity. Error bars represent between subject standard errors for each condition.

The results of Experiment 1 are shown in Figure 4. A 3 × 2 repeated measures factorial analysis of variance (ANOVA) was used to compare JNDs derived under static distractor conditions to those measured in dynamic distractor conditions (effect of *distractor context*) across the three target disparity conditions (effect of *target disparity*). Mauchly’s test of sphericity indicated that the homogeneity of variance for difference scores between pairs of repeated levels assumption was met for all conditions.

With α set at .05, the main effect of *distractor context* was significant, *F*(1, 12) = 14.885, *p* = .002, η_p_^2^ = .554, indicating that overall, dynamic distractors produced significant threshold elevation relative to static distractor conditions. Across observers, the static distractor condition yielded an average JND of 24.8 ms (standard error = 0.8 ms) with the dynamic distractor context yielding an average JND of 199.8 ms (standard error = 59.1 ms). A significant main effect of *target disparity* was observed, *F*(2, 12) = 7.434, *p* = .003, η_p_^2^ = .480. The *target disparity* by *distractor context* interaction term was found to be statistically significant, *F*(2, 12) = 7.226, *p* = .003, η_p_^2 ^= .456. Figure 3(d) plots this interaction.

To deconstruct the significant interaction term, a simple effects analysis and then three simple comparisons were conducted. First, in order to determine whether *target disparity* effects were exclusive to dynamic contextual conditions a simple effects analysis of static contextual conditions was conducted via a one-way ANOVA. The simple effect for static contextual conditions was nonsignificant, *F*(2, 12) = .088, *p* = .916, indicating that the disparity conditions had no effect in static distractor conditions.

Given these findings, we compared the effects of each *target disparity* condition in the dynamic distractor condition. Three paired samples *t* tests were used to conduct the simple comparison analysis. Bonferroni adjustments were performed for each simple comparison in order to maintain a family-wise error rate at .05. The simple comparison between crossed and zero disparity at dynamic distractors was found to be significant, *t*(12) = −3.137, *p* = .009. The simple comparison comparing uncrossed and zero disparity at dynamic distractors was found to be nonsignificant, *t*(12) = −1.143, *p* = .275. A further simple comparison between static and dynamic distractor contextual conditions for crossed disparity was utilised in order to determine whether crossed disparity was sufficient to completely relieve the RTC effect. However, the *t* test revealed a significant context effect, *t*(12) = −3.480, *p* = .005, indicating that performance was significantly worse in the dynamic condition compared with the static condition.

Overall, these simple comparisons indicate that the threshold elevation observed in the zero disparity condition in the presence of dynamic distractors was less severe when the targets possess crossed disparity but not uncrossed disparity.

Experiment 1 showed that the mere presence of dynamic distractor elements impaired the temporal precision with which observers were able to perform TOJs, replicating the RTC effect (Cass & Van der Burg, 2014). The stimulus configuration of Experiment 1 differed from the original study by Cass and Van der Burg (2014) in several respects, including the use of vertically arranged target distractor arrays rather than horizontal arrays, and fewer distractors (four distractors surrounding each target rather than 10). That the magnitude of threshold elevation was on average approximately twice that of the original RTC study (Cass & Van der Burg, 2014) speaks to the robustness and strength of this phenomenon.

Experiment 1 partially confirmed our second hypothesis showing that the magnitude of threshold elevation due to dynamic distractors *can* be reduced by introducing binocular disparity to the target object. This supports previous literature showing that introducing disparity differences between a target and its surrounding context can reduce performance-diminishing contextual effects (Astle-et al., 2014; Felisberti, Solomon, & Morgan, 2005; Kooi et al., 1994; Wardle et al., 2010).

Curiously, in Experiment 1, threshold elevation was significantly reduced *only* in the crossed disparity condition, with no performance benefit provided by uncrossed disparity targets. That performance depends upon the sign of target disparity contrasts findings by Kooi et al. (1994), and Wardle et al. (2010), both of whom reported similar performance benefits using crossed and uncrossed target disparities embedded in otherwise deleterious visual contexts presented with zero disparity. Other studies investigating the effects of target disparity on contextual visual performance, however, echo our asymmetric target disparity effects (Astle et al., 2014; Felisberti et al., 2005; Harris & Morgan, 1993).

What might account for this asymmetry? One intriguing possibility is that it reflects a processing advantage for near relative to far visual objects (Landers & Cormack, 1997). That is to say, performance improves when targets appear in a closer depth plane than its contextual surround compared with situations in which the target is perceived in the same or a more distant depth plane. This asymmetry is echoed in electrophysiological evidence indicating contextual suppression of primary visual cortical neurons can be alleviated by presenting the suppressive contextual stimulus in a more distant (disparity defined) depth plane that the stimulus centred on the cells’ classical receptive field (Sugita, 1999). Additionally, an inherent difference in fusion limits presents a potential contributing factor for the observed asymmetrical disparity effects. Prior research has shown that disparity fusion tolerance is greater for crossed than for uncrossed disparities (Yeh & Silverstein, 1990). Given that the perceived disparity generated by the stimulus in this study was quite large (800 arcsec), it is possible that some observers may have experienced a greater incidence of target diplopia in the uncrossed condition relative to the crossed condition. Such an asymmetry would adversely impact performance in the dynamic distractor condition due to increased likelihood of source confusions between targets and nearby distractors. The observed asymmetry could also reflect a variance in attentional capacities, as prior studies have also demonstrated that observers have an attentional bias toward objects that are perceptually closer (Andersen & Kramer, 1993; Gawryszewski, Riggio, Rizzolatti, & Umiltá, 1987).

## Experiment 2

Experiment 2 aimed to examine whether the reduction in performance for dynamic distractors in the crossed disparity target condition in Experiment 1 is specific to the crossed disparity targets—and therefore possibly the result of fusional problems in the uncrossed target disparity condition, or whether depth order (and hence relative disparity) between target and distractors is a critical factor. In Experiment 2, we manipulate the disparity (crossed, zero, and uncrossed) of the distractors instead of the target (Experiment 1). We expect that if the results in Experiment 1 are due to depth order, then Experiment 2 should produce analogous asymmetric results, with crossed distractors the sole disparity condition expected to reduce threshold elevation.

### Results and Discussion

**Figure 5.**
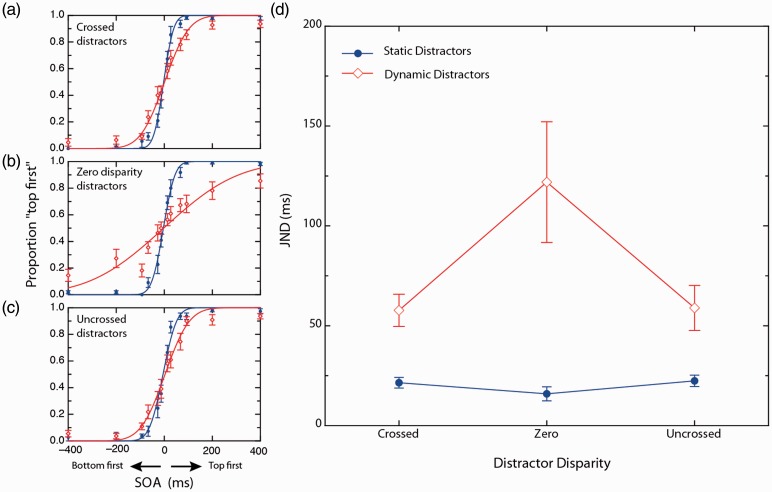
(a) to (c) Effect of distractor dynamics on performance at each level of target SOA averaged across observers. Red and blue symbols and curves are measured under dynamic and static distractor conditions, respectively. (a) Crossed disparity distractor array, (b) zero disparity distractor array, and (c) uncrossed disparity distractor array. Curves shown are cumulative Gaussian fits of observer-averaged data and are shown here for illustrative purposes only. Note that targets were fixed at zero disparity. Statistical analyses were based on fits of individual observers. (d) Mean JNDs derived under static and dynamic contextual conditions, for crossed, zero, and uncrossed distractor disparities. Error bars represent between subject standard errors for each condition.

Analysis of Experiment 2 was identical to that of Experiment 1 except that we manipulated the distractor disparity instead of the target disparity. The main effect of contextual dynamics was significant, *F*(1, 10) = 17.969, *p* = .002, η_p_^2^ = .642, with mean JNDs significantly lower in the static condition (20 ms) relative to the dynamic condition (80 ms). The main effect of disparity was also significant, *F*(2, 10) = 4.911, *p* = .018, η_p_^2^ = .329, indicating that there was a significant difference between at least two of the three levels of disparity when averaged across both levels of distractor contextual condition. The disparity × contextual dynamics interaction was found to be statistically significant, *F*(2, 10) = 5.826, *p* = .010, η_p_^2 ^= .368. Figure 5(d) plots this interaction.

To understand the two-way interaction, a one-way ANOVA assessed the three disparity levels in the static distractor conditions. This was not significant, *F*(2, 10) = 1.118, *p* = .340, indicating that the significant interaction between distractor disparity and distractor dynamics was due to significant disparity effects in the dynamic distractor conditions alone. An examination of Figure 5(d) suggests a reduction in the magnitude of RTC in both conditions involving dynamic distractor disparity (crossed and uncrossed), compared with the zero disparity condition. A two-tailed *t* tests yielded no reliable difference between crossed and uncrossed dynamic distractor conditions, *t*(10) = 0.124, *p* = .904. However, a significant difference was observed between the dynamic zero disparity and dynamic crossed, *t*(10) = 2.462, *p* = .034; and dynamic uncrossed distractor conditions, *t*(10) = 2.364, *p* = .040.

These symmetric distractor disparity effects contrast with the asymmetric target disparity effects in Experiment 1. A potential explanation for this inconsistency could lie in differences in individual fusion limits. In Experiment 1, performance was poorer when the targets were presented in uncrossed disparity. However, in Experiment 2, both targets were presented at fixation in both nonzero disparity conditions.

## Experiment 3

This experiment aimed to further determine whether target or distractor disparity differences *per se* are sufficient to improve TOJ thresholds under dynamic contextual conditions by targets presented with different signs of disparity (e.g., one target presented in crossed disparity, the other uncrossed). In Experiment 3, we therefore manipulate the disparity of each target separately, while keeping the distractors at zero disparity. It is hypothesised that if disparity differences between targets and distractors alone are sufficient to reduce RTC, then we should observe reductions in thresholds when each target is presented with opposite signs of disparity relative to the zero target disparity condition.

### Results and Discussion

**Figure 6.**
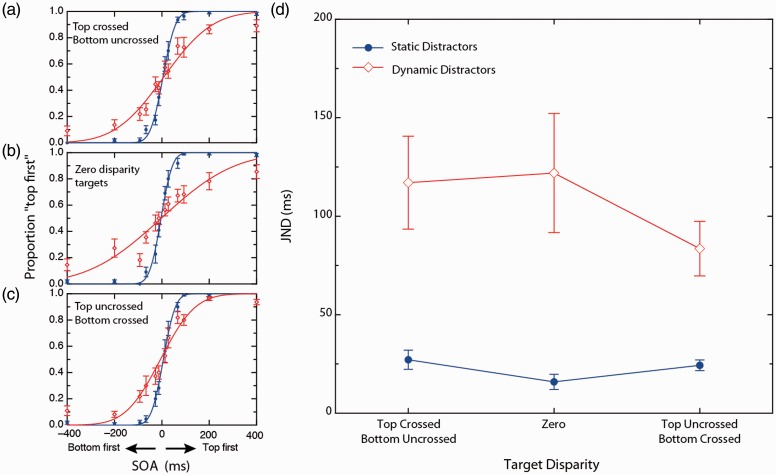
(a) to (c) Effect of distractor disparity and dynamics on performance at each level of target SOA averaged across observers. Red and blue symbols and curves are measured under dynamic and static distractor conditions, respectively. (a) Top crossed, bottom uncrossed disparity targets, (b) zero disparity targets, and (c) top uncrossed, bottom crossed disparity targets. Curves shown are cumulative Gaussian fits of observer-averaged data and are shown here for illustrative purposes only. Statistical analyses were based on fits of individual observers. (d) Mean JNDs derived under static and dynamic contextual conditions, for top crossed bottom uncrossed, zero, and top uncrossed bottom crossed levels of target disparity. Note distractors were fixed at zero disparity. Error bars represent between subject standard errors for each condition.

The main effect of distractor dynamics was significant, *F*(1, 10) = 22.134, *p* = .001, η_p_^2 ^= .689, indicating that there was a significant difference between on JNDs between the effects of static and dynamic distractors when averaged across the three levels of disparity. Means for static context and dynamic context were 22 ms and 108 ms, respectively. Thus, JNDs were significantly higher in the dynamic contextual conditions compared with the static contextual conditions. The main effect of disparity was nonsignificant, *F*(2, 10) = 1.162, *p* = .333, η_p_^2 ^= .104, indicating that there was no significant difference between any of the three levels of disparity when averaged across static and dynamic distractors. The disparity by distractor interaction term was found to be nonsignificant (Figure 6), *F*(2, 10) = 1.774, *p* = .195, η_p_^2 ^= .151.

These results show that presenting each target in a different depth plane fails to relieve the deleterious effects of dynamic distractor environments (RTC). This implies that the disparity-linked improvements in performance observed in Experiments 1 and 2 were not due to the targets simply being presented in different depth planes to dynamic distractors.

## General Discussion

In all three experiments temporally cluttered distractor environments profoundly impaired TOJ acuity performance relative to static distractor conditions, replicating the RTC effect (Cass & Van der Burg, 2014). This study demonstrates that under dynamically cluttered visual conditions, presenting targets and distractors in different disparity-defined depth planes can significantly improve temporal order acuity performance.

Curiously, we find RTC to stronger overall than in Cass and Van der Burg’s original study, despite the current study using fewer distractor elements (8 vs. 20). Whilst we have not conducted a systematic manipulation of the number of distractor items we suspect that the global orientation of the target-distractor array may be responsible, with arrays presented along the vertical meridian in the current study, and the horizontal meridian in the case of Cass and Van der Burg (2014). Indeed, we have presented evidence previously showing that RTC is significantly stronger for TOJ judgments along the vertical relative to the horizontal meridian, possibly implicating attentional factors in RTC (Cass, Gunatillake, & Van der Burg, 2014).

Experiment 1 showed that assigning identical disparities to both targets (crossed or uncrossed) presented in combination with zero-disparity distractors significantly reduced the otherwise severe temporal acuity loss observed when targets and distractors were presented in the same depth plane. This disparity-linked improvement in performance was not symmetric with respect to disparity sign, however, with benefits isolated to the crossed-disparity target condition. This performance asymmetry appears at odds with studies by Kooi et al. (1994), and Wardle et al. (2010) who reported that contextual interference is reduced to an approximately equal magnitude regardless of the sign of target disparity (with contextual stimuli presented at zero disparity). It echoes other work by Astle et al., (2014), however, who show reduced visual crowding (i.e., signifying better performance) from crossed disparity targets relative to uncrossed targets.

Experiment 2 was designed to test two things. First, to determine whether the disparity-linked reductions in RTC observed in Experiment 1 were necessarily linked to the absolute disparity of the target objects or whether assigning disparity to the distractors (and not the targets) might produce similar improvements in performance. Second, Experiment 2 was designed to evaluate the possibility that the asymmetric disparity effects observed in Experiment 1 might signify a depth ordering rule favouring perceptual performance for target events perceived as being closer to the observer than more distant distractor arrays. To examine both sets of questions, we restricted the targets to the zero disparity depth plane and systematically manipulated distractor disparity (crossed, zero, and uncrossed).

The results of Experiment 2 demonstrate that assigning disparity to distractors rather than the targets significantly reduces the magnitude of RTC when targets and distractors are both presented in the same (zero disparity) depth plane. This implies that the disparity-linked improvements in performance observed in Experiment 1 are not necessarily linked to the absolute disparity of the target objects. Rather, it appears that the presence of relative disparity signals between target and distractor objects may be more critical than absolute target disparity for yielding performance improvements in temporally cluttered environments.

That said, the performance improvements in Experiment 2 were not limited to a particular sign of disparity. That is to say, performance improvement was symmetric with respect to distractor disparity. If the instances of performance improvement observed in Experiments 1 and 2 were purely determined by relative disparity signals, then we would expect to observe a performance asymmetry in Experiment 2 that preserved the depth order relations defined in Experiment 1, that is, better performance in the uncrossed distractor condition than the crossed. That we observe asymmetric disparity effects in Experiment 1 but symmetric effects in Experiment 2 suggests the involvement of some other factor(s).

One possible explanation for the asymmetric performance observed in Experiment 1, but not in Experiment 2, may be down to binocular fusion being more reliable for crossed relative to uncrossed targets for this particular group of observers (Howard & Rogers, 1995). That is to say, if the subjects in Experiment 1 found fusing uncrossed targets more difficult than the crossed targets, one would not expect to observe the benefits of disparity afforded by the more reliable crossed disparity target signals. Future research is necessary to determine whether individual differences in crossed and uncrossed fusion limits correlate with our task.

Experiment 3 sought to determine whether assigning each target to a different depth plane (crossed and uncrossed) can reduce threshold elevation caused by dynamic distractors in the zero disparity plane. The results of this experiment show that adding disparity signals of opposite sign to each target does not improve TOJ performance relative to zero disparity target conditions. Assuming that anisotropic fusion is not responsible (as discussed with respect to Experiment 1 earlier), this pattern of results implies the operation of a surface grouping principle, which states that the relative timing of events can be extracted with greater resolution if these events are uniquely located upon a single fronto-parallel plane. What processes or mechanisms might mediate this surface-based principle is unknown.

One possibility is that targets comprising a single unique fronto-parallel surface may promote attentional efficiency, either toward the targets themselves or possibly enabling observers to better ignore distractors in irrelevant depth planes—an idea analogous to visual search performance in the colour domain (Kaptein, Theeuwes, & Van der Heijden, 1995; Van der Burg, Cass, Theeuwes, & Alais, 2015). It is notable that improvements in attentional efficiency have been found to conform to geometrically similar surface-based constraints to those found here (He & Nakayama, 1995; Nakayama & Silverman, 1986).

The spatiotemporal displacement in the onset of each target in our TOJ task introduces lateral motion energy, which can be interpreted perceptually as apparent motion. Assuming that visual TOJs rely on the response of long-range direction-selective motion mechanisms, it seems likely that dynamic distractors might stimulate these same target-informative direction-selective receptive fields. The mere presence of these dynamic distractors therefore predictably introduces noise to the overall decision regarding apparent direction of target-related motion (in the direction opposite to their temporal order), thereby elevating thresholds. An interesting consideration that can be derived from our observed interactions between disparity and RTC is the potential role of area middle temporal (MT) in processing of TOJs (long-range apparent motion). Neurophysiological evidence suggests that visual area MT houses neurons selectively tuned to for both motion direction and absolute disparity (DeAngelis & Newsome, 1999; DeAngelis & Uka, 2003; Maunsell & Van Essen, 1983; Neri, Bridge, & Heeger, 2004; Newsome & Pare, 1988). Whilst, future research might consider MT as a potential candidate for RTC, our inference that RTC appears to be modulated by relative rather than absolute disparity (Experiments 1 and 2) appears to preclude MT as a possible neural substrate for RTC (Neri et al., 2004).

More likely perhaps is that the mechanisms upon which temporal order decisions are based may not be equivalent in static and dynamic distractor conditions. In all experiments, the JNDs associated with static contexts were consistently around 20 to 25 ms, translating to temporal frequencies of ∼40 to 50 Hz. These estimates closely correspond to Holcombe’s *fast* cluster of visual tasks, which incorporates first-order motion discrimination (Holcombe, 2009). By contrast, JNDs in the dynamic distractor conditions were between ∼90 and 250 ms (∼4–11 Hz), corresponding with Holcombe’s *slow* task cluster. Interestingly, a functional hallmark of tasks constituting this *slow* cluster is their reliance on high-level processes such as visual attentional tracking (i.e., high-level motion; Holcombe & Cavanagh, 2001; VanRullen, 2016). We propose, therefore, that whereas TOJ performance in temporally uncluttered conditions (e.g., isolated targets or static distractors) is informed by low-level long-range motion mechanisms, the presence of temporal clutter may force one to rely on high-level motion tracking mechanisms.

Differentiating TOJ performance in this way may serve to explain why disparity effects were only observed in our dynamic—not static—distractor conditions. Attentionally demanding visual search tasks are highly dependent upon the three-dimensional layout of the scene (He & Nakayama, 1995; Nakayama & Silverman, 1986). Assuming that TOJs in our dynamic distractor conditions involve high-level attentional tracking mechanisms, it is perhaps unsurprising that performance in these conditions alone should be sensitive to disparity-defined segmentation principles—that is, partial release from RTC when targets form a single unique coplanar surface.

## Conclusion

To summarise, visual TOJ thresholds are profoundly elevated by the mere presence of irrelevant dynamic events elsewhere in the visual field. The magnitude of this threshold elevation is contingent upon the relative disparity of target and distractor events. That is to say, disparity can be used to alleviate the performance-diminishing effects of RTC but only if both targets constitute a single coplanar surface distinct from the distractors.
